# Tilted-Mode All-Optical Diffractive Deep Neural Networks

**DOI:** 10.3390/mi16010008

**Published:** 2024-12-25

**Authors:** Mingzhu Song, Xuhui Zhuang, Lu Rong, Junsheng Wang

**Affiliations:** 1 Liaoning Key Laboratory of Marine Sensing and Intelligent Detection, Dalian Maritime University, Dalian 116026, China; songmz@dlmu.edu.cn (M.S.); izhuangxh@dlmu.edu.cn (X.Z.); 2 Department of Information Science and Technology, Dalian Maritime University, Dalian 116026, China; 3 School of Physics and Optoelectronic Engineering, Beijing University of Technology, Beijing 100124, China; ronglu@bjut.edu.cn

**Keywords:** diffractive deep neural networks, computing imaging, optical neural networks

## Abstract

Diffractive deep neural networks (D^2^NNs) typically adopt a densely cascaded arrangement of diffractive masks, leading to multiple reflections of diffracted light between adjacent masks, thereby affecting the network’s inference capability. It is challenging to fully simulate this multiple-reflection phenomenon. To eliminate this phenomenon, we designed tilted-mode all-optical diffractive deep neural networks (T-D^2^NNs) and proposed a theoretical model for diffraction propagation in the tilted mode. Simulation results indicate that T-D^2^NNs address the performance degradation caused by interlayer reflections in D^2^NNs constructed with high-index diffractive masks. In classification tasks, T-D^2^NNs achieve better classification results compared to D^2^NNs that consider interlayer reflections.

## 1. Introduction

Artificial neural networks (ANNs) have advanced significantly in various fields [1], including image recognition [2,3], inverse imaging [4,5], and optical holography [6,7]. The computational requirements of ANNs have grown exponentially, owing to the increasing complexity of their application. Electronic chips based on the von Neumann architecture face challenges such as low information processing rates and heating issues during prolonged usage. These challenges primarily arise from the limitations of the stored-program architecture and thermal effects, which hamper the chip performance [8,9]. These challenges make it difficult for electronic chips to satisfy the computational demands of artificial neural networks. Photonic computing, which inherently integrates computation and memory, offers significantly higher information processing rates [10]. It also provides advantages such as high-speed computation, high parallelism, low power consumption, and low latency, making it an emerging focus for constructing ANNs [11,12]. With the advancement of materials science and nano-photonics, researchers have begun to apply photonic computing techniques to the construction of ANNs. For example, Shen et al. demonstrated on-chip, coherent, optical neural morphological computation for vowel recognition datasets by integrating several programmable Mach–Zehnder interferometers on a silicon photonics platform, achieving a classification accuracy comparable to traditional digital computation [13]. Lin et al. constructed a fully optical diffraction deep neural network (D^2^NN) by tightly cascading multiple passive diffraction layers and accurately classifying handwritten digits in the MNIST dataset in both experimental and simulation scenarios [14].

Subsequent simulation and experimental studies on the D^2^NN have enhanced its learning capabilities, particularly in handling complex datasets [15] and multi-task classification [16]. However, researchers have observed a decrease in the accuracy between the experimental and simulated results, which is often attributed to imperfect experimental designs [14]. Furthermore, the D^2^NN typically adopts a densely cascaded arrangement of diffraction masks, which leads to multiple reflections of diffracted light between adjacent masks. Lou et al. pointed out that, for materials with low refractive indices, the reflection effect can be ignored. However, for materials with high refractive indices, the reflection effect significantly affects the results, leading to a non-negligible decrease in the classification accuracy between the experimental and simulated results [17]. In addition, it is challenging to fully simulate the multilayer reflection phenomenon between the diffractive masks in computational simulations.

This study aimed to eliminate multiple reflections between diffraction layers by designing tilted-mode all-optical diffractive deep neural networks (T-D^2^NNs). We proposed a theoretical model for diffraction propagation under tilted incidence and evaluated the performance of T-D^2^NNs and D^2^NNs (considering interlayer reflections) constructed with low-index or high-index diffractive masks, as classifiers on the MNIST and Fashion-MNIST datasets. The results indicate that for low-index diffractive masks, T-D^2^NNs’ performance is like D^2^NNs, albeit with slightly lower accuracy. However, for high-index diffractive masks, T-D^2^NNs outperform D^2^NNs.

## 2. The Structure of the D^2^NN

The D^2^NN consists of multiple passive diffractive masks stacked at specific intervals. Each pixel in every diffractive mask is considered as a “neuron”. During the computer training, iterative adjustments were made to the amplitude and phase of each neuron. Once the training is completed, neurons are mapped onto every diffractive mask using 3D printing or lithography.

**Figure 1:**
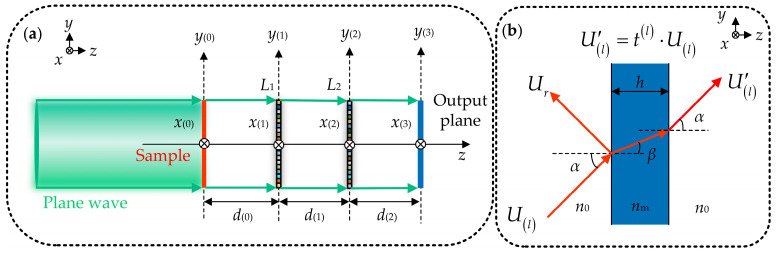
(**a**) The D^2^NN contains two-layers of diffractive masks; (**b**) model of the light wave transmission process through each small pixel.

To illustrate the optical wave propagation process within D^2^NN, for simplicity, we assume that the D^2^NN has two diffractive masks. As shown in Figure 1a, the input layer, diffraction masks, and output layer are positioned on the (*x*_(*i*)_, *y*_(*i*)_) plane, *i* = {0, 1, 2, 3}, with the light beam vertically incident along the *z*-axis on D^2^NN. The complex amplitude distributions of the light field on the input plane, the front and back surfaces of the first and second layer diffractive masks, and the output plane can be represented as $U_{\left(0\right)}^{\prime }$(*x*_(0)_, *y*_(0)_, *z*), *U*_(1)_(*x*_(1)_, *y*_(1)_, *z*), $U_{\left(1\right)}^{\prime }$(*x*_(1)_, *y*_(1)_, *z*), *U*_(2)_(*x*_(2)_, *y*_(2)_, *z*), $U_{\left(2\right)}^{\prime }$(*x*_(2)_, *y*_(2)_, *z*), *U*_(3)_(*x*_(3)_, *y*_(3)_, *z*). According to Rayleigh–Sommerfeld diffraction theory, *U*_(*l*)_ (*x*_(*l*)_, *y*_(*l*)_, *z*), *l* = {1, 2}, can be expressed as:

(1) $$\begin{array}{c} U_{\left(l\right)}\left(x_{\left(l\right)},y_{\left(l\right)},z\right)=\iint_{\infty }{U^{\prime }}_{\left(l-1\right)}\left(x_{\left(l-1\right)},y_{\left(l-1\right)},z\right)\left[\frac{d_{\left(l-1\right)}}{r_{\left(l-1\right)}^{2}}\left(\frac{1}{j\lambda }\right.\right.+\left.\left.\frac{1}{2\pi r_{\left(l-1\right)}}\right)\right]\\ \times \exp\left(j\frac{2\pi r_{\left(l-1\right)}}{\lambda }\right)dx_{\left(l-1\right)}dy_{\left(l-1\right)},\\ r_{\left(l-1\right)}=\sqrt{{\left(x_{\left(l\right)}-x_{\left(l-1\right)}\right)}^{2}+{\left(y_{\left(l\right)}-y_{\left(l-1\right)}\right)}^{2}+d_{\left(l-1\right)}^{2}}, \end{array}$$

where *d*_(*l*−1)_ represents the axial spacing along the *z*-axis from the (*l* − 1)-th layer diffractive mask to the (*l*)-th layer diffractive mask, and λ is the wavelength of the light wave. As shown in Figure 1b, when the light wave passes through the neurons on the (*l*)-th layer diffractive mask with thickness varying with position, pixel-level phase modulation is achieved. The transmission coefficient of neurons on the (*l*)-th layer of diffraction can be expressed as [18,19,20]:

(2) $$t^{\left(l\right)}=\frac{4p_{1}p_{2}\exp\left[jk\left(p_{2}-p_{1}\right)h^{\left(l\right)}\left(x_{\left(i\right)},y_{\left(i\right)}\right)\right]}{{\left(p_{1}+p_{2}\right)}^{2}-{\left(p_{1}-p_{2}\right)}^{2}\exp\left[j2kh^{\left(l\right)}\left(x_{\left(i\right)},y_{\left(i\right)}\right)p_{2}\right]},$$

where *h*^(*l*)^(*x*_(*i*)_, *y*_(*i*)_) represents the geometrical height of the (*i*)-th pixel in the (*l*)-th diffraction mask, *h*^(*l*)^ ∈ [0, λ/*n_m_*), *p*_1_ = *n*_0_∙cos*α*, *α* ∈ [0, π/2), *p*_2_ = *n_m_*∙cos*β*, *k* = 2π/λ, *α* is the angle of incidence, and *β* is the angle of refraction. When the incident light is vertically incident, *α* is 0. When *U*_(*l*)_(*x*_(*l*)_, *y*_(*l*)_, *z*) is modulated by the neurons in the (*l*)-th layer diffraction mask, **$U_{\left(l\right)}^{\prime }$** (*x*_(*l*)_, *y*_(*l*)_, *z*) can be expressed as:

(3) $${U^{\prime }}_{\left(l\right)}\left(x_{\left(l\right)},y_{\left(l\right)},z\right)=U_{\left(l\right)}\left(x_{\left(l\right)},y_{\left(l\right)},z\right)\cdot t^{\left(l\right)},$$

where *t* ^(*l*)^ is given by Equation (2). The forward light propagation process is illustrated in Figure 1a and can be expressed as:

(4) $${U^{\prime }}_{\left(0\right)}\to U_{\left(1\right)}\to {U^{\prime }}_{\left(1\right)}\to U_{\left(2\right)}\to {U^{\prime }}_{\left(2\right)}\to U_{\left(3\right)}.$$

Furthermore, based on the error between the output and expected values, backpropagation is performed to optimize the geometrical height of each pixel in the diffraction masks.

## 3. The Structure of T-D^2^NN

**Figure 2:**
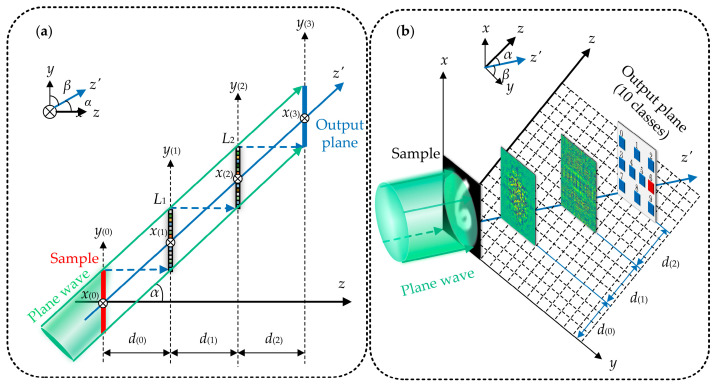
(**a**) This T-D^2^NN contains two-layers of diffractive masks, and the tilt angle of the incident light is α. (**b**) Classifier for handwritten digits.

To eliminate the multiple reflections between the diffraction masks, we designed a tilted-mode network structure. For simplicity, to illustrate the optical wave propagation process within the T-D^2^NN, we assume a T-D^2^NN with two layers of diffractive masks, as shown in Figure 2. Figure 2a,b show the two-dimensional and three-dimensional structural diagrams of a T-D^2^NN with two diffraction masks, respectively. Furthermore, $U_{\left(0\right)}^{\prime }$(*x*_(0)_, *y*_(0)_, *z*) can be represented as [21]:

(5) $${U^{\prime }}_{\left(0\right)}\left(x_{\left(0\right)},y_{\left(0\right)},z\right)=O_{\left(0\right)}\left(x_{\left(0\right)},y_{\left(0\right)},z\right)\exp\left[jk\left(y_{\left(0\right)}\sin a\right.\right.\left.\left.+z\cos a\right)\right],$$

where *O*_(0)_ (*x*_(0)_, *y*_(0)_, *z*) represents the complex amplitude distribution of the incident light when vertically incident. To ensure that the diffracted light from the input plane to the first layer of the diffractive masks does not reflect to the input plane upon reflection, *α* must satisfy the following condition:

(6) $$\tan a\geq \frac{N\cdot d_{f}}{d_{\left(l-1\right)}},$$

where *N* represents the number of neurons per row or column in each diffraction layer, *d_f_* represents the physical side length of each diffraction unit within the diffraction layer, and *d*_(*l*−1)_ represents the axial spacing along the *z*-axis from the (*l* − 1)-th layer diffractive mask to the (*l*)-th layer diffractive mask, *l* = {1, 2}.

According to Rayleigh–Sommerfeld diffraction theory, *U*_(*l*)_ (*x*_(*l*)_, *y*_(*l*)_, *z*) can be expressed as:

(7) $$\begin{array}{c} U_{\left(l\right)}\left(x_{\left(l\right)},y_{\left(l\right)},z\right)=\iint_{\infty }{U^{\prime }}_{\left(l-1\right)}\left(x_{\left(l-1\right)},y_{\left(l-1\right)},z\right)\left(\frac{d_{\left(l-1\right)}\cos\alpha +\sqrt{r_{\left(l-1\right)}^{2}-d_{\left(l-1\right)}^{2}}\sin\alpha }{r_{\left(l-1\right)}^{2}}\right),\\ \times \left(\frac{1}{j\lambda }\right.+\left.\frac{1}{2\pi r_{\left(l-1\right)}}\right)\exp\left(j\frac{2\pi r_{\left(l-1\right)}}{\lambda }\right)dx_{\left(l-1\right)}dy_{\left(l-1\right)}\\ r_{\left(l-1\right)}=\sqrt{{\left(x_{\left(l\right)}-x_{\left(l-1\right)}\right)}^{2}+{\left(y_{\left(l\right)}-y_{\left(l-1\right)}\right)}^{2}+d_{\left(l-1\right)}^{2}}, \end{array}$$

where *y*_(*l*)_ = *y*_(*l*−1)_ + *d*_(*l*−1)_∙ tan*α*. When the diffraction of light satisfies the Fresnel approximation condition, *U*_(*l*)_ (*x*_(*l*)_, *y*_(*l*)_, *z*) can be expressed as:

(8) $$\begin{array}{c} U_{\left(l\right)}\left(x_{\left(l\right)},y_{\left(l\right)},z\right)=\frac{\exp\left[jk\left(d_{\left(l-1\right)}/\cos a\right)\right]}{d_{\left(l-1\right)}/\cos a}\iint_{\infty }{U^{\prime }}_{\left(l-1\right)}\left(x_{\left(l-1\right)},y_{\left(l-1\right)},z\right)\exp\left\{j\frac{k}{2d_{\left(l-1\right)}/\cos a}\right.\\ \times \left[\cos^{2}a{\left(x_{\left(l\right)}-x_{\left(l-1\right)}\right)}^{2}\right.\left.\left.+{\left(y_{\left(l\right)}-y_{\left(l-1\right)}\right)}^{2}\right]\right\}dx_{\left(l-1\right)}dy_{\left(l-1\right)} \end{array}.$$

The optical field distribution at the back surface of the (*l*)-th layer diffractive mask is given by Equation (3). Furthermore, the forward light propagation process shown in Figure 2 can be expressed using Equation (4). The geometrical height of each pixel in the diffraction masks can be optimized by backpropagation.

## 4. The Impact of Interlayer Reflection on the D^2^NN

**Figure 3:**
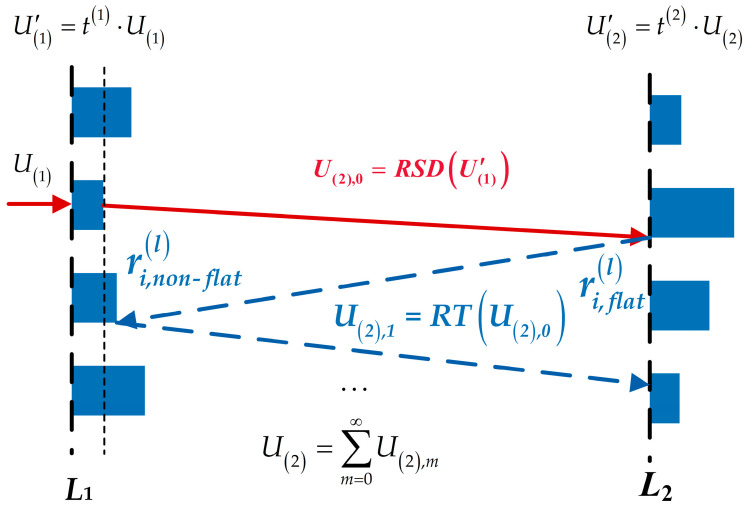
Interlayer reflection effect.

The reflected optical field is expressed as the product of the incident optical field and the reflection coefficient at each pixel position. The input and output planes for the reflection coefficient are defined as two wavefront planes with a 2π phase difference. As illustrated in Figure 3, for the (*l*)-th diffractive layer, the field reflection coefficient at the *i*-th pixel on the aligned side and the field transmission coefficient on the opposite side can be expressed, respectively, as [18,19,20]:

(9) $$\begin{array}{c} r_{i,flat}^{\left(l\right)}=\frac{(1-n_{m}^{2})\left\{1-\exp\left[2kn_{m}h^{\left(l\right)}\left(x_{\left(i\right)},y_{\left(i\right)}\right)\right]\right\}}{{\left(1+n_{m}\right)}^{2}-{\left(1-n_{m}\right)}^{2}\exp\left[j2kn_{m}h^{\left(l\right)}\left(x_{\left(i\right)},y_{\left(i\right)}\right)\right]},\\ r_{i,nonflat}^{\left(l\right)}=\frac{(1-n_{m}^{2})\left\{\exp\left[-j2kh^{\left(l\right)}\left(x_{\left(i\right)},y_{\left(i\right)}\right)\right]-\exp\left[2k(n_{m}-1)h^{\left(l\right)}\left(x_{\left(i\right)},y_{\left(i\right)}\right)\right]\right\}}{{\left(1+n_{m}\right)}^{2}-{\left(1-n_{m}\right)}^{2}\exp\left[j2kn_{m}h^{\left(l\right)}\left(x_{\left(i\right)},y_{\left(i\right)}\right)\right]}. \end{array}$$

As indicated by Equation (9), the magnitude of the reflection coefficient increases with the refractive index. A higher reflection coefficient results in a greater proportion of reflected light, leading to stronger multiple reflections between adjacent diffractive layers, thereby exerting a more significant impact on the overall performance of the D^2^NN. As shown in Figure 3, the total incident field distribution *U*_(2)_ on second diffractive layer from the output field $U_{\left(1\right)}^{\prime }$ of the first diffractive layer can be expressed as:

(10) $$\begin{array}{c} U_{\left(2\right)}=\sum_{m=0}^{\infty }U_{(2),m},\\ U_{(2),0}=RSD({U^{\prime }}_{\left(1\right)}),\\ \forall m>0,U_{(2),m}=RT(U_{(2),m-1}),\\ RT(U)=RSD\left\{RSD\left\{U\cdot r_{flat}^{\left(l\right)}\right\}\cdot r_{non-flat}^{\left(l-1\right)}\right\}, \end{array}$$

where *RSD* and *RT* represent the Rayleigh–Sommerfeld diffraction operator and the round-trip propagation operator between two diffractive layers, respectively.

To evaluate the impact of interlayer reflections on the performance of the D^2^NN, we conducted an image classification task using the D^2^NN. The parameters of the D^2^NN are summarized in Table 1 and Table 2. Additionally, we used the sigmoid function to constrain the pixel height to the range of [0, λ/*n_m_*). The network implementation was based on Python 3.8.0 and Pytorch 1.12.1, running on a laptop equipped with an Intel i7-12700H CPU @ 2.7GHz, 16.0G RAM, NVIDIA GeForce RTX 3060 Laptop GPU, and the Microsoft Windows 11 operating system.

The experiments used the MNIST and Fashion-MNIST datasets with images upscaled prior to experimentation to match the network dimensions. For the MNIST dataset [22], 50,000 images were used for training, 10,000 images for validation, and 10,000 images for testing. Similarly, for the Fashion-MNIST dataset [23], 50,000 images were used for training, 10,000 images for validation, and 10,000 images for testing. The classification accuracy is defined as the total number of correct predictions to the total number of predictions.

**Figure 4:**
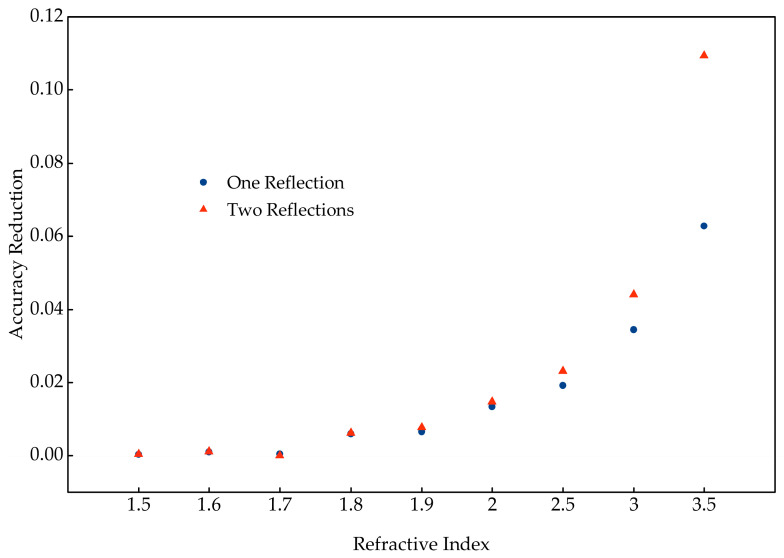
Classification accuracy reductions between D^2^NNs without interlayer reflections and those considering one or two interlayer reflections, for diffractive masks with varying refractive indices.

To quantify the effects of interlayer reflections on D^2^NNs’ performance, we selected five types of diffractive masks with refractive indices of 1.5, 2.0, 2.5, 3.0, and 3.5 to construct D^2^NNs for testing. Through simulations, we investigated the effects of one or two interlayer reflections between diffractive masks with different refractive indices on D^2^NNs’ classification accuracy. Figure 4 shows the effects of one or two interlayer reflections on the performance of D^2^NNs constructed using diffractive masks with varying refractive indices when applied to the MNIST dataset, with the horizontal axis representing the refractive index and the vertical axis showing the reduction in classification accuracy.

As shown in Figure 4, the reduction in classification accuracy caused by one or two interlayer reflections becomes progressively larger with increasing refractive index. When the refractive index is 1.5, the accuracy reduction is negligible. When the refractive index is 2.0, the accuracy reduction cannot be ignored. Additionally, as the refractive index increases, the effects become significantly more pronounced. To determine at which refractive index the reduction in classification accuracy due to interlayer reflections becomes significant, we further simulated the impact of one or two interlayer reflections on the classification accuracy of D^2^NNs using diffractive masks with refractive indices ranging from 1.5 to 2.0, with increments of 0.1. When the refractive index is 1.8, the classification accuracy of the D^2^NN considering one interlayer reflection is reduced by 0.006 compared to a D^2^NN without considering interlayer reflection. At refractive indices of 1.8, 1.9, 2.0, 2.5, 3.0, and 3.5, increasing the number of interlayer reflections further exacerbates the reduction in classification accuracy. Additionally, the higher the refractive index, the more pronounced this reduction becomes. When the refractive index reaches 3.5, one interlayer reflection results in a drop of 0.06 in classification accuracy, while two reflections cause a drop of 0.11.

**Figure 5:**
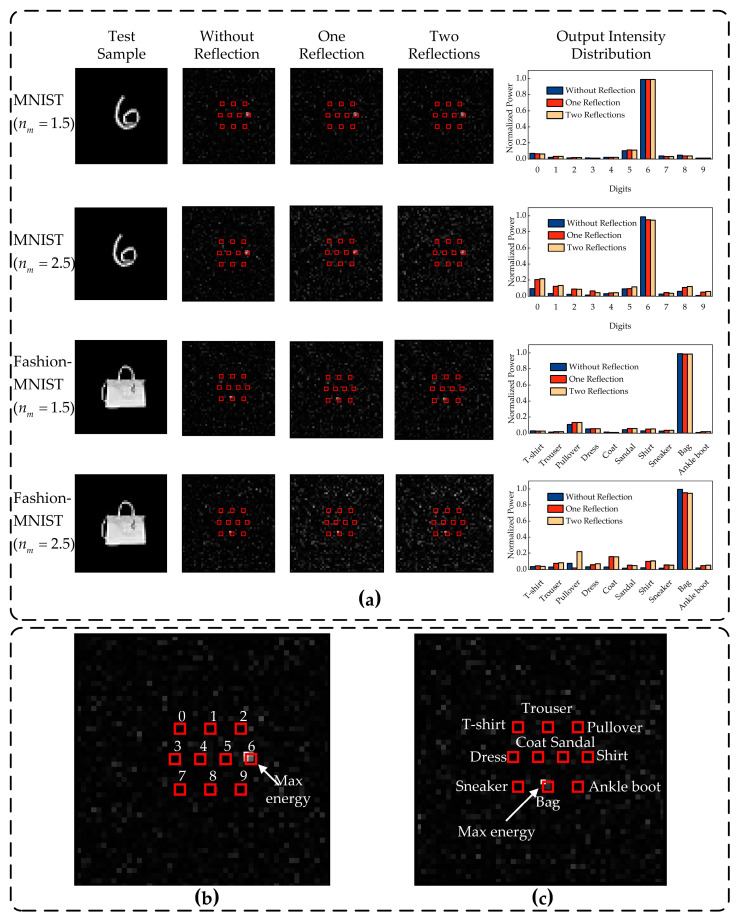
Verification of the impact of varying the number of inter-layer reflections on D2NNs at different refractive indices. (**a**) The intensity distribution at the detector layer for test samples from the MNIST and Fashion-MNIST datasets, considering one or two reflections between diffractive masks at refractive indices of 1.5 and 2.5. (**b**,**c**) present enlarged and annotated views of the intensity distributions at the detector layer for test samples from the MNIST and Fashion-MNIST datasets, assuming no reflections occur between the diffractive masks, which have a refractive index of 1.5.

Figure 5a shows the intensity distribution at the detector layer for test samples from the MNIST and Fashion-MNIST datasets, considering one or two reflections between diffractive masks at refractive indices of 1.5 and 2.5. Figure 5b,c shows the enlarged and annotated views of the intensity distributions at the detector layer for test samples from the MNIST and Fashion-MNIST datasets, assuming no reflections occur between the diffractive masks, which have a refractive index of 1.5. As shown in Figure 5a, compared to the case without interlayer reflections, the effects of one or two interlayer reflections on the intensity distribution at the detector layer are minimal when the refractive index is 1.5. However, at a refractive index of 2.5, the effects result in increased light scattering into non-target regions. Furthermore, the last image in each row shows that, when considering the reflection effects, the intensity of the target class is noticeably lower than in the case without reflections, while the intensity of other classes is significantly higher.

In summary, based on the experimental results, we defined a threshold refractive index of 1.8 to classify the effects of interlayer reflection on network performance. Scenarios with a refractive index below 1.8 were defined as “low refractive index”, while those with a refractive index of 1.8 or higher were defined as “high refractive index”. It is important to note that this classification is a relative concept used to facilitate discussions in Section 4 and Section 5. For D^2^NNs constructed with low-index diffractive masks, the reflection effects have minimal impact on the network’s learning ability. Conversely, for D^2^NNs constructed with high-index diffractive masks, the negative effects of reflections increase significantly as the refractive index rises, and the effects on the network’s learning capacity become more pronounced. Based on the simulation results and theoretical analysis, we infer that this is primarily because high refractive indices correspond to higher reflection coefficients, causing a significant portion of light traveling from one layer to the next to be reflected. After multiple interlayer reflections, these effects can severely impair the network’s learning ability. In contrast, for low refractive indices, the reflection coefficients are relatively small, resulting in only a minor fraction of light being reflected, thus having a negligible impact on the network’s performance.

## 5. Performance Evaluation

To validate the effectiveness of the proposed T-D^2^NNs, we conducted image classification tasks using both D^2^NNs (considering interlayer reflections) and T-D^2^NNs. The D^2^NNs mentioned in this section all consider interlayer reflections.

For T-D^2^NNs, their parameters are referenced in Table 1 and Table 2. According to Equation (6), the tilt angle should be greater than 33.5°. Therefore, we set the tilt angle to 35° in this section. Under this configuration, the axial spacing of the tilted-mode network along the z-direction is 40λ. To ensure that the propagation distance between layers in both D^2^NNs and T-D^2^NNs remains consistent, we set the interlayer spacing for D^2^NNs to 40λ/cos (35°). This arrangement ensures that D^2^NNs satisfy the fully connected condition and that the diffracted light from each small pixel in T-D^2^NNs can cover all pixels in the next diffractive layer, achieving a fully connected effect like that of D^2^NNs. Other network parameters for D^2^NNs are also referenced from Table 1 and Table 2.

To analyze the effects of reflections between diffractive masks, we assumed that only two reflections occur between the diffractive masks in D^2^NNs (referred to as TRD^2^NNs). We simulated the performance of T-D^2^NNs and TRD^2^NNs, both constructed with low-index or high-index diffractive masks, as classifiers on the MNIST and Fashion-MNIST datasets. For the low-index diffractive masks, a refractive index of 1.5 (e.g., IP-S, a commonly used commercial photoresist) was adopted, while for the high-index diffractive masks, a value of 2.58 (e.g., TiO₂) was used. Table 3 shows the classification accuracies of T-D^2^NNs and TRD^2^NNs on the MNIST and Fashion-MNIST datasets for these two refractive index conditions.

As shown in Table 3, for the high-index diffractive masks, T-D^2^NNs effectively improve classification accuracy compared to TRD^2^NNs. For example, in the MNIST dataset, the classification accuracy increases by 0.0156, and in the Fashion-MNIST dataset, it increases by 0.0191. In contrast, for low-index diffractive masks, the classification performance of T-D^2^NNs is slightly lower than that of TRD^2^NNs, but the difference is minimal.

**Figure 6:**
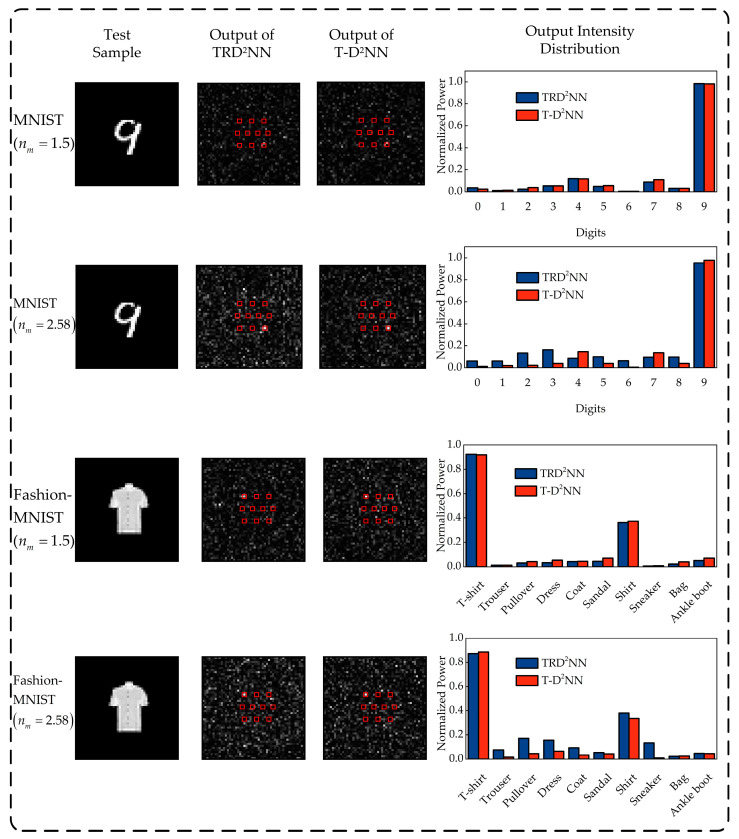
The performance of MNIST and Fashion-MNIST test samples was evaluated using both TRD^2^NNs and T-D^2^NNs with diffractive masks at refractive indices of 1.5 and 2.58.

Additionally, as shown in Figure 6, we show the performance of MNIST and Fashion-MNIST test samples in both TRD^2^NNs and T-D^2^NNs. As shown in the first and third rows of Figure 6, under low-index diffractive masks, the intensity distributions on the output plane are largely similar for both TRD^2^NNs and T-D^2^NNs. In contrast, as shown in the second and fourth rows of Figure 6, for high-index diffractive masks, the intensity distribution at the detector plane is more focused after passing through T-D^2^NNs, with less light scattered to non-target areas compared to TRD^2^NNs.

In summary, the performances of TRD^2^NNs and T-D^2^NNs with low-index diffractive masks are nearly identical. For high-index diffractive masks, T-D^2^NNs outperform TRD^2^NNs. Based on the simulation results and theoretical analysis, we infer that this is primarily due to the weak interlayer reflections in TRD^2^NNs constructed with low-index diffractive masks, which have minimal impacts on network performance. Although T-D^2^NNs can suppress these reflections, the effect is negligible due to the inherently weak reflections. Additionally, because T-D^2^NNs have higher computational complexity and more challenging parameter optimization compared to TRD^2^NNs, they may experience gradient vanishing issues during training. As a result, the performance of T-D^2^NNs constructed with low-index diffractive masks is slightly lower than TRD^2^NNs, but the difference is minor. In contrast, for TRD^2^NNs constructed with high-index diffractive masks, interlayer reflections are strong and significantly degrade network performance. T-D^2^NNs effectively mitigate these reflections, leading to superior performance compared to TRD^2^NNs.

Additionally, we evaluated the performance variations in T-D^2^NNs under different angles. We selected four incident angles of 35°, 45°, 55°, and 65°, with the refractive index fixed at 2.58, while other network parameters remained unchanged, as referenced in Table 1 and Table 2. For the MNIST dataset, Table 4 presents the classification accuracies for these four incident angles.

As shown in Table 4, the classification accuracies of T-D^2^NNs decrease progressively with increasing incident angles. Based on the simulation results and theoretical analysis, we infer that this is primarily due to the increased diffraction losses caused by larger incident angles, which reduce the network’s learning capacity.

## 6. Conclusions

D^2^NNs typically adopt a densely cascaded arrangement of diffractive masks, leading to multiple reflections of diffracted light between adjacent masks, thereby affecting the network’s inference capability. Accurately simulating this multiple-reflection phenomenon is challenging. To address this issue, we propose T-D^2^NNs and introduce a theoretical model for diffraction propagation under tilted incidence. Compared with D^2^NNs, T-D^2^NNs adjust the arrangement of diffractive masks, preventing the diffracted light from the preceding mask from being reflected after reaching the subsequent mask.

We analyzed the effects of interlayer reflections on the performance of D^2^NNs and found that, for networks constructed with low-index diffractive masks, interlayer reflections have a minimal impact on network performance. In contrast, for networks constructed with high-index diffractive masks, network performance significantly degrades as the refractive index and the number of interlayer reflections increase. Based on the simulation results and theoretical analysis, we infer that, for networks constructed with high-index diffractive masks, strong interlayer reflections lead to a pronounced decline in performance due to high reflection coefficients. Conversely, for networks constructed with low-index diffractive masks, the reflection coefficients are small, resulting in weak reflection effects and, thus, minimal impacts on network performance.

We further compared T-D^2^NNs and D^2^NNs (considering interlayer reflections). We assumed that only two reflections occur between the diffractive masks in D^2^NNs (referred to as TRD^2^NNs). We simulated the performance of T-D^2^NNs and TRD^2^NNs, both constructed with low-index and high-index diffractive masks, as classifiers on the MNIST and Fashion-MNIST datasets. The results show that, for low-index diffractive masks, the performance of T-D^2^NNs is comparable to TRD^2^NNs, with T-D^2^NNs being slightly inferior. In contrast, for high-index diffractive masks, T-D^2^NNs outperform TRD^2^NNs, achieving classification accuracy improvements of 0.0156 and 0.0191 for the MNIST and Fashion-MNIST datasets, respectively. Based on the simulation results and theoretical analysis, we infer that, for TRD^2^NNs constructed with low-index diffractive masks, interlayer reflections are weak and have only minor effects on network performance. Although T-D^2^NNs suppress interlayer reflections, the effects are negligible due to the inherently weak reflection effects. Additionally, T-D^2^NNs have a higher computational complexity, making parameter optimization more challenging compared to TRD^2^NNs, which can result in gradient vanishing during training. Therefore, the performance of T-D^2^NNs slightly decreases but remains close to TRD^2^NNs. For TRD^2^NNs constructed with high-index diffractive masks, strong interlayer reflections significantly degrade performance, while T-D^2^NNs demonstrate superior classification performance by mitigating these reflections. We also evaluated the classification performance of T-D^2^NNs under different incident angles. Based on the simulation results and theoretical analysis, we infer that as the incident angle increases, the performance of T-D^2^NNs gradually decreases. This is primarily due to increased diffraction losses at larger angles, which adversely affect the network performance.

This method may offer new insights for experimental verification in manufacturing, real-time imaging, super-resolution imaging, optical holography, image enhancement, and classification tasks using D^2^NNs with high-refractive-index materials. Additionally, experimental verification of the feasibility and accuracy of the theoretical model will be a future research direction.

## Figures and Tables

**Table 1:** Structure Parameters of D^2^NN.

Parameter	Value
Illumination wavelength (λ)	632.8 nm
Diffraction unit size (*d_f_*)	0.53λ
Layer distance (*d*_(0)_, …, *d*_(5)_)	40λ
Number of diffractive layers (*l*)	5
Unit number on each layer (*N*×*N*)	50 × 50
Refractive index of the material (*n_m_*)	{1.5, 2.0, 2.5, 3.0, 3.5}

**Table 2:** Optimization Parameters of D^2^NN.

Parameter	Value
Loss function	MSE
Optimizer	Adam
Learning rate	0.002
Number of diffractive layers (*l*)	5
Batch number	200
Epoch number	85

**Table 3:** Classification accuracy of D^2^NNs, T-D^2^NNs and TRD^2^NNs under various refractive index conditions.

Refractive Index	MNIST		Fashion-MNIST	
	TRD^2^NN	T-D^2^NN	TRD^2^NN	T-D^2^NN
1.5	0.9299	0.9257	0.8504	0.8453
2.58	0.9098	0.9254	0.8246	0.8437

**Table 4:** Classification accuracies of T-D^2^NNs on the MNIST dataset during training at various incident angles.

Incident Angle	35°	45°	55°	65°
Classification Accuracy	0.9254	0.9229	0.9211	0.9202

## Data Availability

Data are contained within the article.
